# An Account of the Practice of One of the Physicians of the Westminster General Dispensary, and of the Western Dispensary, from the 20th of January, to the 20th of February, 1807

**Published:** 1807-03

**Authors:** Samuel Fothergill

**Affiliations:** Southampton Street, Strand


### *An Account of the Practice of one of the Physicians of the Westminster General Dispensary, and of the Western Dispensary, from the 20th of January, to the 20th of February*, 1807.

| ACUTE DISEASES. | | | |
|---|---|---|---|
| Catarrh | 8 | Pleurisy | 1 |
| Peripneumony | 1 | Angina | 2 |
| | | Acute Rheumatism | 3 |

Continued

| | |
|---|---|
| Continued Fever - | 2 |
| Erysipelas - - | 1 |
| Hooping Cough - | 3 |
| Enteritis - - - | 1 |
| Acute Diseases of Infants | 8 |
| Periodical Diseases. | |
| Quotidian - - | 1 |
| Tic Douloureux - | 1 |
| CHRONIC DISEASES. | |
| Asthenia - - - | 10 |
| Pulmonary Consumption | 5 |
| Hæmoptoe - - - | 3 |
| Cough and Dyspnœa - | 34 |
| Asthma - - - | 4 |
| Peripneumonia Notha | 3 |
| Pleurodyne - - | 3 |
| Chronic Rheumatism - | 5 |
| Gout - - - | 2 |
| Cephalalgia - - | 2 |
| Epilepsy - - - | 1 |
| Apoplexy - - - | 2 |

| | |
|---|---|
| Hydrocephalus - - | 1 |
| Paralysis - - - | 5 |
| Tabes Mesenterica - | 2 |
| Scrophula - - | 3 |
| Worms - - - | 2 |
| Gastrodynia - - | 8 |
| Hæmatemesis - - | 1 |
| Dyspepsia - - | 8 |
| Colic - - - | 2 |
| Diarrhœa - - - | 5 |
| Dysure - - - | 2 |
| Jaundice - - - | 3 |
| Hæmorrhoids - - | 1 |
| Hydrothorax - - | 1 |
| Anasarca - - - | 3 |
| Syphilis - - - | 2 |
| Cutaneous Diseases - | 7 |
| Amenorrhœa - - | 6 |
| Menorrhagia - - | 2 |
| Chlorosis - - - | 3 |
| Leucorrhœa - - | 2 |

Though the season has hitherto been generally mild, considerable and sudden changes of temperature, with some stormy weather, have been experienced. Coughs, with catarrhal and pulmonic affections, continue to be the prevailing complaints, and in some instances have been severe, accompanied with pleuritic pains, hæmoptoe, and dyspnœa. Many people, advanced in years, have been carried off; in some, the thread of life has suddenly snapped, whilst others have yielded to long continued complaints, which winter renders more difficult to be resisted.

Whenever the bodily powers are decayed, or the vigor of the vital functions is permanently diminished, the constitution must be considered as aged, though the years of the patient be not numerous; and whether premature old age be effected by early excess, or is occasioned by severe and frequent attacks of disease, the consequences are to be feared, and the feeble frame to be supported with assiduous and *continual* care, for when once the habit of precaution is adopted, to deviate is often fatal. By a guarded mode of living, using early hours, uniform temperance, and warmth in clothing, avoiding the night air, fatigue, and long fasting, a very crazy constitution is frequently piloted into old age, through the severity of succeeding winters and the perils of disease.

If

If it be important to shield the aged from the rigour of winter, and guard them against the effects of debility, it is not less necessary to foster the tenderness of infancy. Infants and young children, in London, are very susceptible of disease, and not seldom perish from the want of *early* medical attendance. This observation, perhaps, is more correct when applied to the poor: yet, from some instances within my notice, it is applicable also to the higher ranks of society. A superficial knowledge of medicine is now become pretty general, and many amiable and excellent women with a medicine chest, do more mischief than is generally imagined, though it is not difficult to conceive, that with an engine so potent, where the will is good, much may be accomplished; and at length we are reluctantly called in to witness a most miserable misapplication of medicine. Nothing but magnesia and rhubarb should be entrusted to *amateur* practitioners.

Another cause of death to children is, their mothers having heard that Nature will do every thing, and that to disturb her operations is a busy, interested interference, only productive of evil; they view their child daily sinking under a lingering disease, or they mark with astonishment the rapid march of croup or of peripneumony, *hoping* the next hour may produce a favourable change, till the child is fairly dead of Nature, certainly not of the Doctor, who in such instances, if sent for at all, can only console himself with having an opportunity of pursuing his studies in morbid anatomy.

Another source of fatality to children is, the over-great tenderness of the mother, who refuses to give the child a dose of calomel, because she has heard that people may be salivated; and will not suffer its head to be shaved, because of the "dear sweet hair." An instance of this sort occurred to my observation very lately. A medical man was called in to a case of acute hydrocephalus, and very properly directed a blister to be applied to the head, mercurial frictions, &c. The mother was astonished, consulted her friends, and sent for a physician, with whom I saw the patient; a day was, however, necessarily lost, and our approbation of the practice which had been previously proposed, did not seem to raise a very favourable opinion of us in the mind of the mother. Upon calling the next day I found the child's head still unshaven, and nothing active done, the parents having too good an opinion of their own judgment to be guided by that of their medical advisers.

SAMUEL FOTHERGILL.

*Southampton Street, Strand.*

